# Virtual Nursing for the Care of Hospitalized Patients

**DOI:** 10.1001/jamanetworkopen.2025.45597

**Published:** 2025-12-05

**Authors:** K. Jane Muir, Alexandra Maye, Matthew D. McHugh, Linda H. Aiken, Vicky Vo, Karen B. Lasater

**Affiliations:** 1 Center for Health Outcomes and Policy Research, School of Nursing, University of Pennsylvania, Philadelphia; 2 The Leonard Davis Institute of Health Economics, University of Pennsylvania, Philadelphia; 3 Department of Emergency Medicine, Perelman School of Medicine, University of Pennsylvania, Philadelphia

## Abstract

**Importance:**

It is unknown if virtual nursing (VN) enhances care quality or improves the workload of bedside nurses in hospitals.

**Objective:**

To describe what services VNs provide to patients and nurses in hospitals; to evaluate whether the presence of VNs improves nurse workload and patient care quality; and to examine bedside nurses’ experiences with VNs.

**Design, Setting, and Participants:**

This cross-sectional study using mixed methods included results from the 2024 Nurses4All survey conducted between December 2023 and March 2024. Eligible respondents were hospital-employed registered nurses in 10 states.

**Exposure:**

Experience providing VN-assisted bedside care.

**Main Outcomes and Measures:**

Bedside nurses reported the services provided by VNs and answered questions about VNs’ impact on workload and quality of care. Nurses also answered the open-text response, “Please share any positive or negative experiences you have had working with virtual nurses.”

**Results:**

The registered nurse sample included 880 respondents with a mean (SD) age of 44.2 (12.3) years and 13.3 (11.3) years of experience (92 Asian [10.5%], 87 Black [9.9%], 514 White [58.4%]; 84 [9.5%] with Hispanic ethnicity). The top uses of VNs reported by nurses were patient observation (434 nurses [53%]), admission and discharge activities (381 nurses [45%]), and patient education (306 nurses [37%]). The majority of nurses (483 nurses [57%]) reported that VNs did not reduce their workload, and among these, 81 nurses (10%) said VNs increased their workload. Of the 366 nurses (43%) who said VNs reduced their workloads, only 70 (8%) reported that workloads were reduced “by a lot.” Slightly over half of nurses (452 [53%]) said VNs improved quality of care, but only 96 (11%) reported that quality was improved “by a lot.” VNs had no impact on care quality according to 391 nurses (47%), with 34 (4%) stating that VNs reduced care quality. Nurses described the strengths of VNs for monitoring and documentation, and limitations related to staffing, patient distrust, and workflow inefficiencies.

**Conclusions and Relevance:**

In this cross-sectional study, there were mixed findings on VNs, suggesting such models should be introduced cautiously.

## Key Points

**Question:**

Are virtual nurses (VNs) in hospitals associated with bedside nurses’ workloads and care quality?

**Findings:**

In this cross-sectional study using mixed methods that included 880 registered nurses, the majority of nurses (57%) reported that VNs did not reduce their workload, and among these 10% said VNs increased their workload. Of the 43% who said VNs reduced workloads, only 8% reported that workloads were reduced “by a lot.” Slightly over half (53%) of nurses said VNs improved quality of care, but only 11% reported that quality was improved “by a lot.” When asked how VNs affect quality of care, 47% of nurses reported no impact on care quality, with 4% reporting that VNs reduced care quality.

**Meaning:**

These findings suggest that virtual nursing yields mixed results in hospitals.

## Teaser

This cross-sectional study of survey responses from US nurses evaluates the contribution of virtual nursing to bedside nurses’ workloads and care quality.

## Introduction

Even before the COVID-19 pandemic, 1 in 2 hospital bedside nurses reported high burnout, 1 in 3 were dissatisfied with their jobs, and 1 in 5 intended to leave their employer within the year.^1^ In the aftermath of the pandemic, hospitals have struggled to recruit and retain bedside nurses with turnover rates in 2024 at 16.4%.^2^ Hospital leaders are exploring alternative nursing care models that increase registered nurse retention, reduce the number of registered nurses (RNs) required at the bedside, or provide additional support to nurses at the bedside without adversely affecting quality of care. Among these alternative care models has been the introduction of virtual nursing (VN; also referred to and abbreviated here as virtual nurses).^3^

VN is a broad term used to define a range of nursing care activities undertaken offsite or in a different location than the inpatient unit—eg, assessment, monitoring, education, care coordination, treatment—that rely on video and/or messaging platforms. The COVID-19 pandemic catapulted the use of VN at the bedside due to patient surges, infection control measures, and nursing care vacancies.^4^ In the postpandemic years, hospital administrators are increasingly considering the expansion of VN care models.^5^ Services and programs that leverage VN in acute general hospital care have included telemetry monitoring, patient admissions and discharges, patient education, and visual monitoring of patients at risk for falls and injuries.^6^

VN in hospitals has been lauded as a novel care model offering the possibility of reducing nurses’ workloads, burnout, and turnover, assisting nurses with patient care tasks, and possibly reducing the number of nurses required to staff hospital inpatient units around the clock.^4,6^ VNs, it is hypothesized, can contribute to care for hospitalized patients remotely, including conducting patient surveillance and assessments, completing documentation tasks (eg, medication reconciliation, review of care plans with family members and patients, ensuring that admission orders are completed), providing patient education, and completing care coordination tasks (eg, discharge planning). VNs also hold promise for the mentorship of bedside nurses, particularly nurses who are new-to-practice. VN may also improve patient experience and quality of care by increasing patients’ interactions with clinicians via the bedside nurse and VN.^4^ Intensive care units have been early adopters of VN, deploying their use to oversee rapid response protocols and expert nurse consultation for medication titration. However, the evidence to date on VN in non–intensive care unit inpatient care has been limited to quality improvement initiatives in single health care systems^5,7,8,9^ and perspectives^10^ and is mixed about whether VN improves care quality.^5,8,9^

There are concerns about the possible adverse impacts of VN on patient outcomes as the majority of hospitalized patients—especially on medical and surgical units—are older and sick, and thus may be challenged by technology and experience hearing, vision, and/or cognitive deficits that could undermine their comprehension via virtual communication methods. Issues around communication between bedside nurses and VNs and the availability of functional technology equipment (eg, audio and video) have also arisen as potential barriers to patient care delivery.^7^ VN could be an add-on without added value unless it is shown to enhance care quality, increase patient safety, reduce nurses’ workload, or improve the well-being and retention of clinical nurses at the bedside. To date, there is limited evidence about the value of VN models on medical and surgical units.

In this study, we analyze data from over 800 bedside nurses working on medical and surgical units in over 400 general acute hospitals in 10 states to understand how VNs are participating in hospital care, and to what extent the care provided by VNs is associated with the workloads of bedside nurses and the quality of patient care.

## Methods

This cross-sectional, sequential mixed-methods study was a descriptive analysis of quantitative and open-text response survey data from nurses. We drew upon the Donabedian model for quality of care^11^ to evaluate how VN is associated with the organization of health care and the processes of care that nurses direct for optimal patient outcomes. The study was approved by the University of Pennsylvania institutional review board. Participants were informed of the survey objectives and provided written consent through their participation in the survey. Data collection, analysis, and reporting of the study findings was conducted in alignment with the Strengthening the Reporting of Observational Studies in Epidemiology (STROBE) and Consolidated Criteria for Reporting Qualitative Research (COREQ) reporting guidelines.

### Data and Sample

Nurses4All is a large survey of nurses who serve as informants about the conditions of their employment setting. Invitations to participate in the survey were emailed to 100% of actively licensed RNs in 9 states (California, Florida, New Jersey, Oregon, Washington, New Mexico, New York, Illinois, Louisiana) and mailed to a random sample of RNs licensed in Pennsylvania. As in prior work,^1,12,13,14^ we followed a modified Dillman survey approach,^15^ and nonrespondents received follow-up reminders at regular intervals. The survey was conducted between December 2023 and March 2024 and yielded over 113 000 responses. The large survey sampling frame reflects the fact that most licensed RNs are not providing direct patient care in hospital settings, but the licensure lists as a sampling frame do not include information on place of employment. Thus, the survey yields responses from nurses in various health care sectors (eg, hospitals, long-term care, school settings, primary care) as well as retired or unemployed nurses who maintained their nursing license. We have shown in prior work employing a criterion standard double-sampling technique that involves intensive nonresponder surveying, that our approach yields little evidence of nonresponse bias.^12^

Germane to the current research question were respondents who identified as RNs employed as direct-care (bedside) staff nurses in hospitals on medical and surgical units, intermediate care (including progressive or step-down care), or hematology-oncology care units and responded to the survey question: “Are there virtual nurses in your workplace (ie, nurses working remotely that can interact with patients/family/team members via video)?” The quantitative survey sample included 880 bedside nurses in 418 hospitals.

The final analytic sample for open-text response analysis included 786 nurses who answered “yes” to the question about whether there are VNs in their workplace, and provided substantive comments to the open-ended, optional survey prompt: “Please share any positive or negative experiences you have had working with VNs.”

### Variables

If hospital nurses answered “yes” to the question about VNs in their workplace, the electronic survey prompted them to select all that apply from a list of services that VNs provided. A response option “other” was provided, with the option to write-in responses. Write-in responses were analyzed and recoded to an existing category when appropriate.

Nurses were asked how much the use of VNs reduced their workload on a 4-point Likert scale (a lot, a little, not at all, VNs increase my workload) and whether the use of VNs improved the quality of care in their practice setting with response options following a 4-point Likert scale (a lot, a little, not at all, VNs reduce the quality of care). In our results reporting, we refer to VNs as reducing nurses’ workload or improving care quality if nurses responded “a lot” or “a little” on the Likert scale of the respective question. Conversely, if nurse workloads or care quality were not improved, this refers to the Likert responses of “not at all” and “virtual nurses increase my workload” or “virtual nurses reduce the quality of care.”

### Data Analysis

Data analysis of quantitative responses used descriptive statistics (frequency tabulations) and data visualization strategies to describe the types of services VNs provide and the extent to which bedside nurses reported the use of VNs impacted care quality and their workloads. Nurse demographics were also analyzed. Nurses were asked to indicate their race and ethnicity in categories predefined in the Nurses4All survey.

Open-text responses were stored and analyzed in NVivo version 15 (Lumivero). Data were scrubbed of identifiers, cleaned, and edited for typos and clarity. Three study authors developed a codebook using deductive concepts from a review of previous research about VN. Examples of initial codes included *workload*, *discharge*, and *safety*. Through open coding of a subset of data, the authors identified subsequent inductive codes to complete the codebook (eg, *monitoring*, *challenges*).^16,17^ Three authors (K.J.M., A.M., V.V.) then coded a second subset of data to resolve discrepancies and achieve an interrater reliability over 0.70 with the codebook. All 3 authors coded the remaining responses.

The research team grouped similar codes into categories, for example: staffing concerns or patient communication. The team iteratively discussed the categories, combining similar categories and deleting categories that were repetitive. When the study team determined that data saturation was achieved, themes were then identified to summarize findings.^18^ The integration between the qualitative and quantitative findings is outlined in eTable in Supplement 1.

To uphold rigor and trustworthiness in the qualitative analytic process,^19,20,21^ a positionality statement was documented reflecting the unique perspectives of each researcher; a decision audit trail was maintained; and the team was comprised of a diverse team of researchers with and without clinical nursing expertise.

## Results

The sample included 880 registered nurses who indicated that VN was used in their workplace. Nurses had a mean (SD) age of 44.2 (12.3) years with 13.3 (11.3) years of experience (11.3); 92 nurses identified as Asian (10.5%), 87 Black (9.9%), and 514 White (58.4%), with 84 (9.5%) reporting Hispanic ethnicity (Table 1). The majority of nurses had at least a Bachelor of Science in Nursing degree (540 nurses [61.4%]). VNs were used for patient observation (454 nurses [53%]), admission/discharge activities (381 [45%]), patient education (306 [37%]) (Table 2). Other less common services provided by VNs included triage, documentation, training or mentoring staff, case management, specialty consultations, preoperative screenings, interpreter services, and serving as a second witness for medication administration.

**Table 1.** Nurse Participant Demographic Information

Characteristic	Nurses, No. (%) (N = 880)^a^
Age, mean (SD), y	44.2 (12.3)
Experience in nursing, mean (SD), y	13.3 (11.3)
Race	
American Indian or Alaskan Native	NR
Asian	92 (10.5)
Black or African American	87 (9.9)
Native Hawaiian or other Pacific Islander	NR
White	514 (58.4)
Multiple races	43 (4.9)
Other race^b^	27 (3.1)
Hispanic ethnicity	
No	704 (80.0)
Yes	84 (9.5)
Education	
BSN or higher	540 (61.4)

Abbreviations: BSN, Bachelor of Science in Nursing; NR, not reported.

^a^ Missing results by variable were: age, 112 nurses; years of experience, 7 nurses; race, 104 nurses; Hispanic ethnicity, 92 nurses; BSN education, 65 nurses. Results not reported were due to small cell size.

^b^ Other included write-in responses from nurses (eg, “multiple race,” “Filipino American”).

**Table 2.** Use of Virtual Nursing in Hospitals

Services	Nurses, No. (%) (N = 880)^a^
Patient observation	454 (53)
Admission and discharge activities	381 (45)
Patient education	306 (37)
Patient documentation	144 (18)
Triage	129 (16)
Training or mentoring staff	109 (13)
Other^b^	167 (20)

^a^ Percentages do not sum to 100% because nurses were instructed to select all that apply. Variables had <7 missing across services that respondents could select.

^b^ Other includes case management, specialty consults, preoperative screenings, interpreter services, and second witnesses for medication administration.

The majority of nurses (483 [57%]) reported that VNs did not reduce their workload, and among these, 81 nurses (10%) said VNs increased their workload (Figure). Of the 366 nurses (43%) who said VNs reduced their workloads, only 70 (8%) said by a lot. Over half of nurses (452 [53%]) said VNs improved quality of care but only 96 (11%) said by a lot. VNs had no impact on care quality as reported by 391 nurses (47%), with 34 (4%) stating that VNs reduced care quality.

**Figure.**
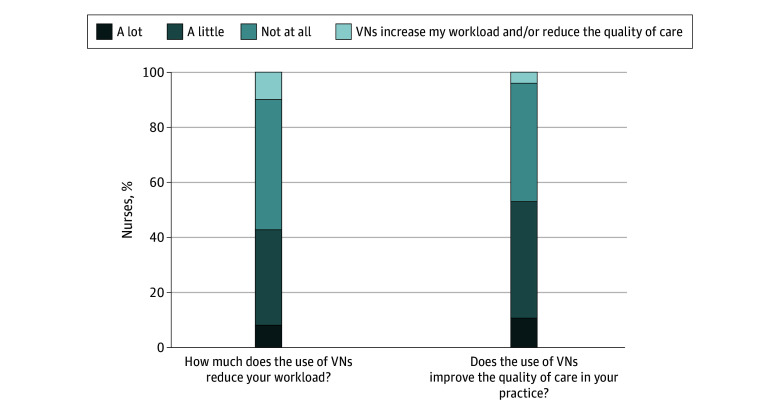
Hospital Nurse Evaluations of Virtual Nursing on Bedside Nurse Workload and Quality of Patient Care

Bedside nurses’ experiences working with VN were described in the following 6 themes: staffing workaround, another pair of eyes, safety risks and time delays, added work, patient distrust, and administrative help or hindrance.

### Staffing Workaround

Bedside nurses described VN as a workaround for inadequate staffing at the bedside (Box). As one medical-surgical nurse stated, “Management is trying to count them in the floor staffing ratio which impacts how much staff we can have based on patient numbers (I would rather give up the virtual nurse in order to have another person on the floor helping with direct patient care).” Another medical-surgical nurse expressed concern that budgeting for bedside nurse staffing was diverted to invest in VNs: “It would be far better to have extra nurses in the hospital actually on the floor able to physically help. VNs just cut into the budget of staffing actual floor nurses on a unit and throughout a hospital.”

Box. **Exemplar Quotes From Thematic Analysis**

**Theme 1: Staffing Workaround**

“We use them instead of using staff as sitters for unsafe/impulsive patients. This is a cost saving measure for the facility but provides less care. Doesn’t seem to be really monitored closely. I know this because we will ask for privacy (per protocol) when providing personal care and frequently get no response.” (Stepdown, progressive care, or intermediate care unit nurse)
“…I feel like all of this is an excuse for administration to leave us at an unsafe patient ratio. Nothing replaces actual hands on the floor to provide direct patient care.” (Medical-surgical nurse)
“I personally would rather have another nurse actually working on the unit to decrease the nurse to patient ratio. I’m guessing we are paying a lot for this service and could use the $$$ for onsite staff.” (Stepdown, progressive care, or intermediate care unit nurse)

**Theme 2: Another Pair of Eyes**

“It can be helpful to have another sets of eyes on the patients but sometimes they do not call fast enough if something is happening with the patient.” (Medical-surgical nurse)
“I find them to be an extra pair of eyes in the care of my patients.” (Stepdown, progressive care, intermediate care unit nurse)

**Theme 3: Safety Risks and Time Delays**

“Patient monitor can get overloaded and patient safety comes to question if they are actually worth the risk versus a sitter which we don’t have.” (Medical-surgical nurse)
“Time consuming, not readily available, delays in patient care.” (Medical-surgical nurse)
“Unfortunately I have not seen the benefit of VNs. …[Other] than a quick warning of a potential problem they seem to function about as well as a bed alarm when a patient is determined to get out of bed.” (Stepdown, progressive care, or intermediate care unit nurse)

**Theme 4: Added Work**

“They can do the admission database to save time, but then you don’t really know your patient. At the end of the day, we are still responsible for our patients so the virtual nurse just adds more to us.” (Medical-surgical nurse)
“Some nurses will call every time a patient moves, others will speak through the 2 way radio to assist. It can be beneficial or a headache.” (Medical-surgical nurse)
“The virtual observer is another pair of eyes for patients who are at a high risk for falls. However, the virtual observer is not available to physically interact with the patient. Quite often, the virtual observer ends up calling the nurse to report a safety issue, and the nurse or other staff must go to manage the situation.” (Medical-surgical nurse)

**Theme 5: Patient Distrust**

“Patients tend to treat them like a commercial during their favorite movie and don’t pay attention to them or try to fast forward them.” (Medical-surgical nurse)
“I only use them on certain floors for admissions. Sometimes they are helpful, but really only for the cooperating, young patient who is NOT in pain, NOT hungry, and has ALL their needs met. Otherwise, the patient’s are too focused on other things and making requests for the staff to do, rather than focusing on an iPad in front of them.” (Stepdown, progressive care, intermediate care unit nurse)
“Frequent patient falls, patient confused and removing medical equipment, patient yelling screaming and attacking staff when in the room. Little live staff assistance to help with these patient issues-step down.” (Stepdown, progressive care, intermediate care unit nurse)

**Theme 6: Administrative Help or Hindrance**

“Saves time completing admissions.” (Medical-surgical nurse)
“They take away from having staff in person. Watching someone on a screen and calling me 50 times is not a substitute for a personal safety attendant or an admission help.” (Stepdown, progressive care, intermediate care unit nurse)
“LOVE that they can help do very time consuming admissions.” (Medical-surgical nurse)

Nurses described preferring more nurses at the bedside over VNs to provide hands-on support and resources. Nurses questioned their employer’s investment in VN when bedside staffing ratios were already suboptimal. One medical-surgical nurse stated: “[VN] does not replace a physical presence to intervene if [the patient is] trying to get up unassisted and we don’t have staff to be in all assigned rooms at one time when many are fall risks. I think administration uses virtual monitoring capability as an excuse not to have enough physical staff on the floor. It is not a substitute.”

### Another Pair of Eyes

VNs were used to observe and monitor patients for potential safety risks such as falls or elopement. A benefit of this resource was an extra pair of eyes to monitor patients. A medical-surgical nurse stated, “I love the fact that a nurse has eyes on a fall risk patient (most likely confused) prone to getting up out of bed.”

VNs identified “good catches,” or preventable safety breaches that may have otherwise compromised patient outcomes. As an intermediate care nurse stated, “They are a good extra set of eyes who can help with documenting. They have even caught bedsides’ errors like IV rates.”

### Safety Risks and Time Delays

Despite VNs’ care surveillance, bedside nurses cited concerns over timeliness in VN communication and patient safety. One medical-surgical nurse stated, “I think a virtual nurse can be helpful if they communicate with the primary nurse but when they do not communicate they can cause an unsafe care environment.” Another medical-surgical nurse expressed concern about VNs being an unreliable safeguard for patient safety: “They are present but do not actually call when safety issues occur.”

### Added Work

Bedside nurses reported about the added work that VNs required of them. VNs created redundant work for bedside nurses who had to fix errors with documentation or take time to explain procedures and policies to VNs. As one medical-surgical nurse stated: “By the time I have communicated a request from the virtual nurse and they have followed through with it (when they had time), it would have taken less time if I just did it myself most of the time. Communicating back and forth for clarification is wasting my time.” Nurses were hesitant to endorse VNs as a time-saving resource. As an intermediate care nurse described, “Frequently it is not effective enough to lessen my workload.”

### Patient Distrust

Nurses stated that patients were distrustful of VNs. One medical-surgical nurse stated, “Floor nurses CONSTANTLY repeat what VNs already taught. Patients lack trust of VNs.” Nurses shared examples of VN fragmenting the patient-clinician relationships. One intermediate care nurse said, “In speaking with a lot of my patients, they consider them to be useless. They ask questions but can’t do anything about it. They then message me concerns or issues that I have to circle back and fix or address.”

### Administrative Help or Hindrance

VNs were helpful in administrative tasks, such as medication reconciliation and hospital admissions and discharges. Nurses endorsed the delegation of lower priority, nonclinical tasks to VNs. Said one medical-surgical nurse, “I use them mostly for discharges and admissions which takes a huge burden off of my workload during the day. They can also do some of the documentation and call report if we are transporting the patient to a rehab or long-term care facility.” One medical-surgical nurse who reported that VNs significantly reduced their workload reported, “I find that VNs remove a lot of documentation burden regarding admission and discharge, so I can be free to settle in patients, do head toe assessments, skin checks, IV [intravenous] line assessment on admission, and help prep patients for discharge in a timely matter.”

For tasks that may be delayed by bedside nurses due to higher acuity care needs, VNs were able to provide support; however, it still required additional follow-up by bedside nurses. Said a medical-surgical nurse, “It helps with admission to the unit, because most of the data is already there, but we still have to do 2 RN skin checks, get the orders fixed and implemented.” One medical-surgical nurse described concerns over gaps in care due to a lack of rapport with VNs: “I do not like using VNs for discharges because they don’t know the patients like the primary RN does. I worry that the virtual nurse will miss something pertinent that I would go over with a patient at discharge. I think utilizing them for discharge leaves gaps in patient care.”

## Discussion

A driving motivation for investments in VN services in hospitals is the expectation that VNs will alleviate bedside nurses’ high workloads^22^ and thus decrease expensive turnover. Our findings on these assumptions are mixed. Over half of bedside nurses in medical and surgical hospital units reported that VNs had either had no impact on their workload or increased it. Quality improvement is another motivating factor in adoption of VN, and over half of nurses reported improved quality associated with VN but only 11% of nurses said VN increased quality by a lot. Close to half of bedside nurses reported the quality of patient care was unchanged, and 4% reported that VN reduced quality of care. Overall, the findings on VNs as a strategy to reduce nurses’ workloads or improve quality are decidedly mixed.

An issue commonly noted by nurses in this study was the investment in VNs in lieu of sufficient nurses at the bedside. This finding is consistent with prior research^23,24^ describing a mismatch between bedside nurses’ and managements’ priorities to advance work environments that support safe patient care and nurse well-being. Over 2 decades of research, including evidence from before, during, and after the COVID-19 pandemic,^23^ demonstrates that patient-to-nurse staffing ratios vary substantially across hospitals with negative consequences for patient safety. In the absence of safe bedside nurse staffing ratios in hospitals, our findings suggest that the implementation of VN programs may be limited because most bedside nurses say they are not associated with improvements to nurses’ workloads.

Investments in hiring more bedside nurses may enhance the contribution of VNs—but not the other way around, as nurses in our study stated. Bedside nurses described VNs as helpful in alleviating administrative tasks that were lower priority for bedside nurses, such as admission and discharge documentation, medication reconciliation, and patient education. However, nurses said that when nurse staffing was inadequate, the marginal benefit of delegating tasks to a virtual nurse was small. This was due in part to hospital nurses needing to double-check the work of the virtual nurse (eg, medication reconciliation lists), resulting in task duplication and workflow inefficiencies.

There are several considerations for VN implementation outlined by leading professional societies as well as 20 years of nursing health services research. The American Nurses Association^25^ has stated that implementation of VN models in hospitals requires outlining specific standards and policies surrounding their use; continued education and training of VN staff; and the provision of VN as a resource to supplement and not replace in-person nurse staffing.

Our findings give little reason for hospitals to be optimistic, all else being equal, that VN is going to solve persistent problems with nurse workload and patient care quality. Hospital leaders should continue to be open to other strategies that have a strong evidence base including improving nurse work environments^26,27,28,29,30^ and investing in nurse staffing adequacy. Prior evidence demonstrates that when nurses work in hospitals with safe patient-to-nurse staffing ratios, patients and nurses experience better outcomes.^31,32,33^

### Limitations

Our analysis was limited to hospital medical-surgical; progression, step-down, and intermediate care; and hematology-oncology care units and is not representative of all hospital units, including intensive care or psychiatric units that may employ VN programs. Study findings cannot be interpreted causally given the cross-sectional and descriptive study design of this investigation. The available data did not permit an analysis of hospital variation in VN implementation.

## Conclusions

We identified mixed findings on the value of virtual nursing in hospitals. Most nurses reported that VNs did not reduce their workload, and slightly over half indicated that VNs improved patient care quality. Bedside nurses cited concerns over added work and care delays associated with the use of VNs. VNs helped with delegation of administrative tasks, but the investment in VNs in the absence of sufficient in-person staffing added more work for nurses at the bedside. Early reports from bedside nurses in 10 states provides little evidence that implementation of VN is a game changer to address the chronic nurse understaffing in hospitals and to improve patient care quality.

## Notes

Supplement 1. eTable. Mixed-Methods Integration

Supplement 2. Data Sharing Statement
