# Supplementary material for: Virtual Nursing for the Care of Hospitalized Patients
Source: JAMA Netw Open. 2025 Dec 5;8(12):e2545597. doi: 10.1001/jamanetworkopen.2025.45597 (PMC12681041; doi:10.1001/jamanetworkopen.2025.45597)
Supplement: Supplement 1. — eTable. Mixed-Methods Integration [file jamanetwopen-e2545597-s001.pdf]

Supplemental Online Content

Muir KJ, Maye A, McHugh MD, Aiken LH, Vo V, Lasater KB. Virtual Nursing for the Care of Hospitalized Patients. *JAMA Netw Open.* 2025;8(11):e2545597. doi:10.1001/jamanetworkopen.2025.45597

eTable. Mixed-Methods Integration

This supplemental material has been provided by the authors to give readers additional information about their work.

© 2025 Muir KJ et al. *JAMA Network Open*.

eTable. Mixed-Methods Integration

Table 3. Pillar Integration of Results				
**Quantitative Data**	**Quantitative Category**	**PILLAR BUILDING THEMES**	**Qualitative Category**	**Qualitative Theme**
Nurses state that virtual nurses reduce their workload "Not at all" (47%) or "Virtual nurses increase my workload" (10%)	Over half of bedside nurses state that the use of virtual nursing either has no impact on their workload or increases it.	Adequate nurse staffing levels are a key indicator of patient care quality and safety and are associated with nurses' job dissatisfaction.	Nurses describe hospital management practices of instituting virtual nursing in the absence of adequate nurse staffing physically at the bedside.	**Staffing workaround**
Virtual nurses were used for patient observation as reported by 53% of bedside nurses	The majority of nurses reported that virtual nurses provide patient observation services.	Additional support to observe patients through virtual nursing is beneficial to reduce potential adverse events and safety risks	The observational services that virtual nursing provide may enhance patient safety	**Another pair of eyes**
Nurses state that virtual nurses improve the quality of patient care "not at all" (43%) or "virtual nurses reduce the quality of care" (4%).	Almost half of bedside nurses reported concerns with patient care quality in the context of virtual nursing.	Virtual nursing can assist in advancing patient safety if nurses are adequately resourced to implement the services.	Nurses reported delays in virtual nursing communicating safety concerns, which exacerbated patient safety risks.	**Safety risks and time delays**

© 2025 Muir KJ et al. *JAMA Network Open*.

Ten percent of bedside nurses report, "Virtual nurses increase my workload"	The implementation of virtual nursing is additive rather than alleviating nurses' workload.	Nurses' resource constraints such as high patient workloads contribute to safety risks.	Nurses experienced added work fixing the work of virtual nurses and responding to excessive alarms that may or may not have been necessary for patient care processes.	**Added work**
Virtual nursing services were used for patient observation (53% of nurses) and patient education 45%).	Virtual nurses provide predominately patient-facing activities.	Patient outcomes depend on high quality relationships and communication between patients and clinicians.	Patients were distrusting virtual nursing due to communication and technological barriers, as well as a hindrance to cultivating high quality relationships with clinicians.	**Patient distrust**
Virtual nursing was used for admission and discharge activities (37% of nurses)	Virtual nurses helped with non-urgent administrative tasks	If tasks are not completed properly, bedside nurses must add that back into their workflow	Virtual nursing helped with administrative tasks but also provided workflow inefficiencies and added work	**Administrative help or hindrance**

Notes: **Data Integration Process:** Integration of the quantitative and qualitative findings was conducted using the Pillar Integration Process, wherein quantitative findings were initially organized in the following matrix with descriptors. Qualitative exemplar quotes were overlayed onto the quantitative findings in the joint display to facilitate thematic analysis.

© 2025 Muir KJ et al. *JAMA Network Open*.
