# Supplementary material for: Virtual Nursing for the Care of Hospitalized Patients
Source: JAMA Netw Open. 2025 Dec 5;8(12):e2545597. doi: 10.1001/jamanetworkopen.2025.45597 (PMC12681041; doi:10.1001/jamanetworkopen.2025.45597)
Supplement: Supplement 2. — Data Sharing Statement [file jamanetwopen-e2545597-s002.pdf]

Data Sharing Statement

Muir. Virtual Nursing for the Care of Hospitalized Patients. *JAMA Netw Open*. Published December 05, 2025. doi:10.1001/jamanetworkopen.2025.45597

Data
Data available: No
